# The Association Between Smoking and Renal Function in People Over 20 Years Old

**DOI:** 10.3389/fmed.2022.870278

**Published:** 2022-06-03

**Authors:** Yi-Cheng Fu, Zhi-Liang Xu, Ming-Yi Zhao, Ke Xu

**Affiliations:** ^1^Department of Paediatrics, Renmin Hospital of Wuhan University, Wuhan University, Wuhan, China; ^2^Department of Paediatrics, The Third Xiangya Hospital, Central South University, Changsha, China; ^3^Xiangya School of Medicine, Central South University, Changsha, China

**Keywords:** smoking, eGFR, chronic kidney disease, NHANES, cotinine, cross-sectional study

## Abstract

**Background:**

Many conclusions have been reached in renal function studies in direct smokers.

**Aim:**

This study aimed to determine the relationship between smoking and decreased renal function to ensure that reduced chronic kidney disease incidence can be achieved by limiting smoking, we assessed the relationship between cigarette smoking and renal function.

**Methods:**

We recruited 10,267 people from the National Health and Nutrition Program Testing Survey (NHANES) aged over 20 years from 2013 to 2018 to assess smoking exposure by serum cotinine. We estimated the glomerular filtration rate (eGFR) and used multivariate linear regression models and smooth curve fittings to assess the relationship between smoking and renal function.

**Results:**

We found an inverse relationship between serum cotinine and the eGFR. In a subgroup analysis, we found a non-linear relationship between serum cotinine and the eGFR in different ethnic groups or in different sexes. In a subgroup analysis of sex, we found inflection points between men and women for the relationship between serum cotinine and the eGFR (men 183 ng/ml and 465 ng/ml; women 227 ng/ml and 412 ng/ml). However, in a subgroup analysis by age, we found that serum cotinine showed a clear negative correlation with the eGFR in people aged 20–39 years, but in people older than 40 years, a weak correlation was shown. In stratified analysis by ethnicity, we found significant negative associations in Mexican American and Other Hispanic individuals and weaker associations in Non-Hispanic White and Non-Hispanic Black individuals.

**Conclusion:**

Through the negative correlation between serum cotinine and the eGFR, we can conclude that as the smoking quantity increases, smoking leads to a decrease in renal function. The results of the subgroup analysis indicate that in young people, by advocating smoking cessation early, we can very effectively prevent kidney disease in this population and thus reduce the incidence of chronic kidney disease. Smoking should be included as an independent risk factor for chronic kidney disease.

## Introduction

Smoking has long been a global health problem, and numerous studies have identified smoking as one of the risk factors for many diseases, including cardiovascular diseases, respiratory diseases, and multiple cancers (1, 2). Since cigarette smoke contains more than 4,000 different substances, the specific mechanisms by which smoking causes various diseases are currently unclear (3). According to relevant *in vitro* studies, smoking may lead to nitric oxide (NO) utilization in the body, thus destroying the antioxidant system. Chemicals in cigarettes can induce an increase in platelet numbers and activation, which can cause abnormal coagulation in smokers, leading to endothelial dysfunction. Smoking can lead to physical damage. Related studies show that cadmium in cigarette smoke can cause vascular endothelial cell death, and these mechanisms interact, eventually causing smoking-related vascular lesions (3–5). However, the mechanisms by which smoking leads to disease are diverse and are associated with the content of chemicals in smoke and the ages of smokers. Some countries are already aware of the harm of smoking and take relevant measures, but a huge population base of smoking still exists worldwide, as well as a large amount of second-hand smoke exposure.

Currently, the number of patients with chronic kidney disease (CKD) is increasing, and CKD has become a global health problem. As a chronic kidney disease, CKD poses great challenges to patient quality of life and survival (6). According to epidemiological surveys, the prevalence rates of CKD in adults aged 20 years and older in 2010 were 10.4% among the population of men and 11.8% among the population of women (7, 8). In the 2016 death factor statistics, deaths of kidney cancer accounted for 32.3% (9). Since CKD is a chronic and irreversible process, identifying and preventing its early risk factors are extremely important.

Cigarettes contain various chemicals (such as cadmium) and have a potential risk of causing kidney injury. The specific mechanism by which smoking damages kidney function is unknown. Currently, known mechanisms involve acute hemodynamics (such as increased blood pressure and glomerular pressure changes) and chronic injury (such as endothelial cell dysfunction). These mechanisms can affect glomerular filtration function and even damage the microvessels of the kidney (10). Although such damage does not occur immediately, over time, damage accumulation will eventually lead to structural changes in the kidney, resulting in kidney disease. The relationship between smoking and kidney injury was confirmed in type 1 diabetes patients in 1979, and smoking was viewed as a major renal risk factor in 1997 (10, 11). Sufficient clinical research evidence indicates that smoking is a risk factor for renal impairment, especially in people with hypertension and type 2 diabetes, which will deteriorate further. In a prospective study, researchers found that in type 2 diabetes, smokers compared with non-smokers, smokers have a significantly increased risk of diabetic nephropathy (12); in Rana Tahbaz et al. researchers found that avoiding smoking can effectively prevent kidney cancer (13), and in recent years, studies have shown that the success rate of renal transplantation was significantly lower for smokers (14–16).

Cotinine is a primary metabolite of nicotine in the human body, which is mainly present in the blood and is a widely used marker of tobacco exposure due to its long half-life (3–4 day) (17). In the current study, cotinine was shown to promote neural excitation and showed some anti-inflammatory effects in some trials. In a study by Valentina Echeverria et al. they found that cotinine has a neuroprotective effect that improves memory and may become a potential drug for Alzheimer’s disease (18). Cotinine metabolism and the advantages and disadvantages of this agent in humans are still debated, and this article uses serum cotinine as a marker of tobacco exposure while exploring the effect of cotinine on nephropathy by identifying the possible relationship between cotinine and the glomerular filtration rate (eGFR).

Relevant studies have shown a link between second-hand smoke exposure and decreased renal function (19, 20), but no progress has been achieved with respect to direct smokers and renal function. To investigate whether smoking can be an independent risk factor for renal decline, we selected 10,267 people from 2013 to 2018 from the National Health and Nutrition Program Testing Survey (NHANES) aged over 20 years, assessed smoking by serum cotinine, and calculated the glomerular filtration rate (eGFR) to try to identify smoking as an independent risk factor for renal decline (21). In the following study, we used multiple linear regression to identify a possible linear relationship between the eGFR and serum cotinine, used smooth curve fitting to verify this relationship, and found inflection points in different populations. The results in different groups rendered our study independent. Our study used a large sample size to explore the relationship between direct smoking and renal function, and by subgroup analysis, we found specific relationships among different populations that had not been found in previous studies.

## Materials and Methods

### Study Population

**FIGURE 1:**
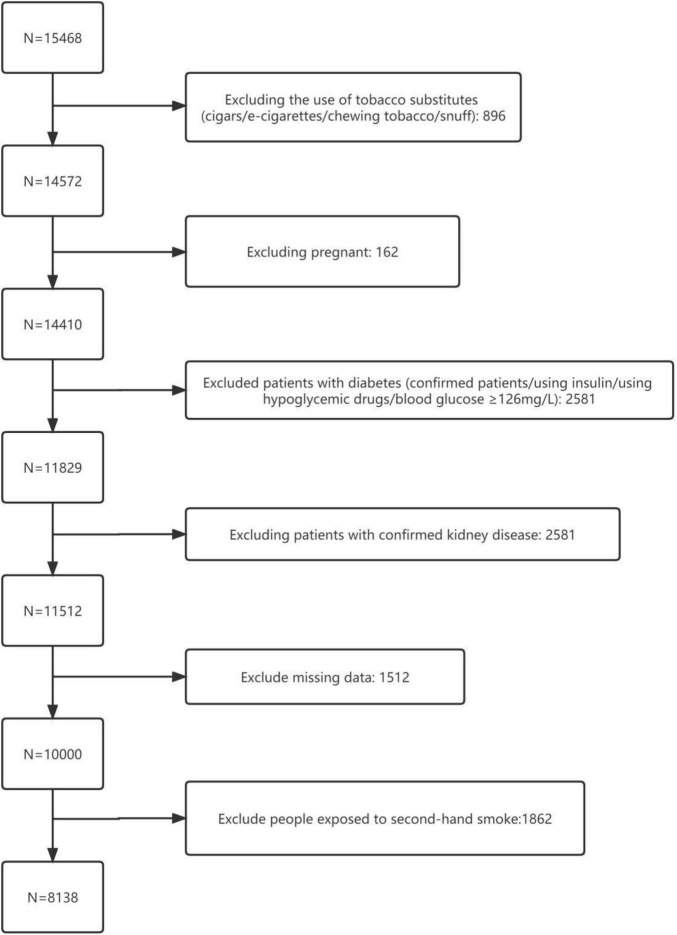
Standard population screening table.

National Health and Nutrition Program Testing Survey is the only national survey that provides a cross-sectional picture of the nutrition and health of the United States population. Data are collected every 2 years by the National Center for Health Statistics (NCHS). For data researchers and users, NHANES survey data are publicly available on the internet. Detailed information about the NHANES can be found at www.cdc.gov/nchs/nhanes/. This study collected data from 2013 to 2018 and included a total of 8,138 eligible participants (Figure 1), including 58,880 current non-smokers (NS) and 2,258 current smokers (CS), and further excluded secondary smoke exposure (SHS) from the form considering cotinine concentrations in an exposure. The NCHS Ethics Review Board approved all of the NHANES protocols, and the participants or their agents provided informed consent prior to their participation.

### Measurements of Smoking

As a metabolite of nicotine, cotinine can be used as a marker of smoking. After referring to relevant literature, we found that Benowitz (22) and others classified smoking as NS (cotinine < 0.05 ng/mL, all races/ethnicity), CS (cotinine 5.92 ng/ml, non-Hispanic black, non-Hispanic whites, and Hispanic populations 4.85 ng/ml and 0.84 ng/ml), and second-hand smokers (SHSers) (non-Hispanic black population 0.05–5.91 ng/ml; non-Hispanic black and non-Hispanic white populations, 0.05–4.84 ng/ml, the Hispanic population, 0.05–0.83 ng/ml). In this study, with no thresholds for Hispanic populations, we used the Mexican American thresholds for Hispanic groups.

### Measures of Renal Function

The serum creatinine-based eGFR was estimated using the modified 4-variable Modification of Diet in Renal Disease (MDRD) Study equation and Chronic Kidney Disease Epidemiology Collaboration (CKD-EPI) equation (23, 24):

eGFR_MDRD_(ml/min/m^2^) = 175 × (Scr)^–1^.^154^ × age^–0^.^203^ × 0.742 (if woman) × 1.212 (if black),

where age is expressed in years and Scr is the standard serum creatinine level in milligrams per deciliter;

eGFR_CKD–EPI_ (mL/min/m^2^) = 141.0 × min (Scr/κ,1)^α^ × max (Scr/κ,1)^–1.209^ × 0.993^age^ × 1.018 (if woman) × 1.159 (if black),

where Scr is the standard serum creatinine, *k* is 0.7 for women and 0.9 for men, α is −0.329 for women and −0.411 for men, and min indicates the minimum of Scr/κ or 1, and max indicates the maximum of Scr/κ or 1.

We classified the participants into the following three eGFR categories: (1) normal renal function with an eGFR ≥ 90 ml/min/m^2^; (2) mildly decreased renal function with an eGFR 60–89 ml/min/m^2^; and (3) moderately to severely decreased renal function with an eGFR < 60 ml/min/m^2^ (6).

### Covariates

For covariates in this study, sex, race/ethnicity, and marital status were used as categorical variables; age, BMI, household income to poverty ratio, blood glucose, glycosylated hemoglobin, BUN, triglycerides, and uric acid were used as continuous variables. In the next studies, we also grouped the continuous variables (age, eGFR values) to obtain more accurate results. During the examination, BMI was calculated based on the measured height and body weight. The ratio of household income to poverty is calculated by dividing household income by the poverty criterion. Details of the covariate acquisition procedure can be found at www.cdc.gov/nchs/nhanes/.

### Statistical Analysis

National Health and Nutrition Program Testing Survey sample weights were calculated in this study. We used a weighted multivariate linear regression model to assess the association of smoking with eGFR values. Correlation covariates were adjusted for potential effect modifiers. In the subgroup analysis, we performed smooth curve fitting to address the non-linearity of cotinine and the eGFR after adjusting for relevant variables. Continuous and categorical variables are expressed as the mean ± SD and percentage, respectively. Data analysis was performed using statistical packages R^1^ and EmpowerStats^2^ Differences were considered statistically significant when the *P*-value was < 0.05.

## Results

A total of 8,138 participants aged 20–80 years were included, with the eGFR as a categorical variable (< 60 ml/min/m^2^, 60–89 mL/min/m^2^, 90–119 ml/min/m^2^, > 120 mL/min/m^2^), as shown in Table 1, where the eGFR was significantly different by age, race, smoking status, and cotinine levels (*P* < 0.0001). We found that sex, race, age, and smoking were significantly associated with the eGFR (*P* < 0.00001). In contrast, we found that women have a greater proportion of renal function decline than men (eGFR < 60 ml/min 85.358% 60 ml/min eGFR < 90 ml/min 66.955); in the racial stratification, we observed that the non-Hispanic populations had lower eGFRs than other races (Non-Hispanic White populations 46.98%; Non-Hispanic Black populations 23.803%). Notably, among the non-Hispanic Black populations, the population with an eGFR < 60 ml/min was higher than that in the other groups; regarding whether a population smokes, by lateral comparison, we found that in the smoking population, the proportion of people with an eGFR < 60 ml/min was higher than that in the other groups (37.764%). Additionally, in the eGFR < 60 ml/min group, serum cotinine levels were significantly higher than those found in the other groups (138.349 ± 201.707), which seems to suggest that cigarette smoking leads to decreased renal function.

**TABLE 1:** Weighted characteristics of the study population stratified based on the eGFR.

	eGFR (< 60 mL/min)	eGFR (60 mL/min ≤, < 90 mL/min)	eGFR (90 mL/min ≤, <120 mL/min)	eGFR (≥ 120 mL/min)	*P*-value
Sex					< 0.00001
Men	14.642	33.045	47.676	44.272	
Women	85.358	66.955	52.324	55.728	
Race/ethnicity					< 0.00001
Mexican American	5.578	2.598	4.869	14.621	
Other Hispanic	2.035	0.58	1.832	12.058	
Non-Hispanic White	46.98	78.512	76.008	56.198	
Non-Hispanic Black	23.803	14.097	9.925	5.349	
Other Race – Including Multi-Racial	21.605	4.213	7.366	11.774	
Age	72.760 ± 7.544	64.879 ± 12.629	51.074 ± 15.058	39.959 ± 14.313	< 0.00001
Educational background					< 0.00001
Less than high school graduate/GED or equivalent	58.034	10.962	8.478	13.874	
High school graduate/GED or equivalent	14.201	20.626	20.104	21.029	
Higher than high school graduate/GED or equivalent	27.765	68.412	71.417	65.098	
Marital status					< 0.00001
Married	38.184	56.445	63.779	54.449	
Spinsterhood or divorced	59.781	40.429	30.4	33.509	
Cohabiting	2.035	3.125	5.82	12.041	
BMI	26.797 ± 4.818	29.222 ± 6.316	28.639 ± 6.181	28.501 ± 6.927	0.0768
Serum cotinine	138.349 ± 201.707	36.501 ± 101.044	55.536 ± 124.071	49.539 ± 109.292	0.0002
Smoking					< 0.00001
No	62.236	83.855	75.993	72.613	
Yes	37.764	16.145	24.007	27.387	

*Mean ± SD for continuous variables: The P-value was calculated by the weighted linear regression model. (%) for categorical variables: the P-value was calculated by the weighted chi-square test. BMI, Body Mass Index.*

**FIGURE 2:**
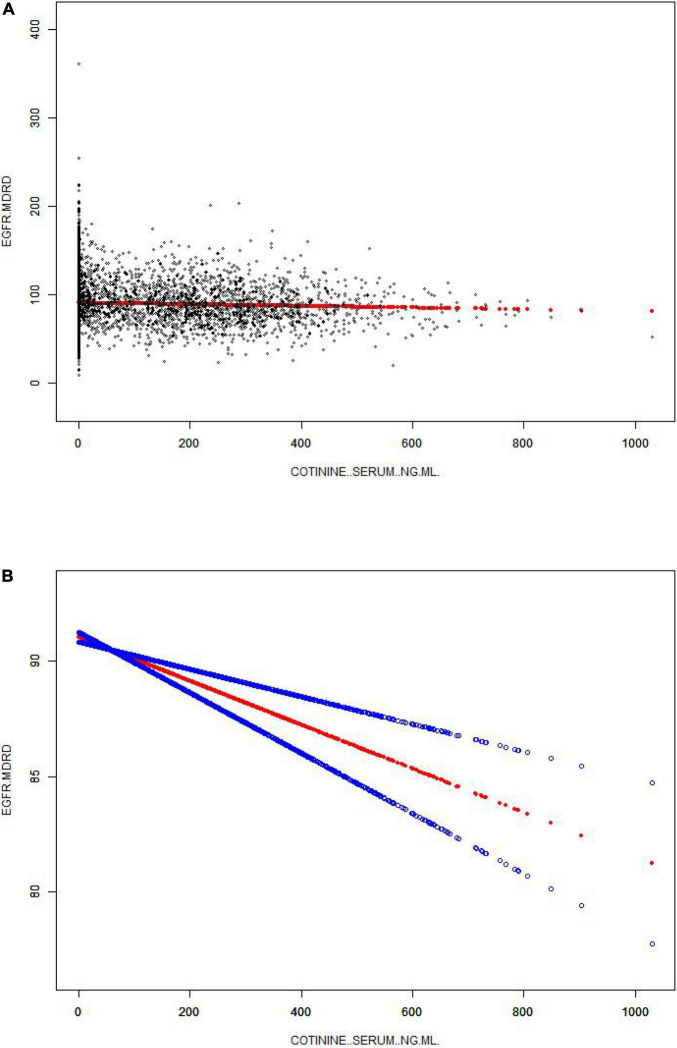
The association between serum cotinine and the eGFR. **(A)** Each black point represents a sample. **(B)** The solid red line represents the smooth curve fit between variables. Blue bands represent the 95% CI from the fit. Age, sex, race/ethnicity, educational background, the ratio of family poverty to income, marital status, BMI, glycosylated hemoglobin, blood glucose, BUN, triglycerides, and uric acid albumin were adjusted.

After fully adjusting the model and controlling for associated confounders, we observed a significant negative correlation between the eGFR and cotinine [−0.0083 (−0.0118, −0.0047)0.000005] (Table 1 and Figure 2) and reached the same conclusion after the smooth curve fitting (Figure 1). By stratifying by sex, age and ethnicity, we found that in the sex stratification, this association remained: men [−0.0048 (−0.0095, −0.0002)0.041537] and women [−0.0112 (−0.0166, −0.0059)0.000039] (Table 2). In the age stratification, in the 20–39-year group, this negative correlation was very obvious [−0.0207 (−0.0275, −0.014) < 0.000001]. In populations older than 40 years, this correlation was absent [−0.0034 (−0.0089, 0.002)0.221484]; [0.0058 (−0.001, 0.0127)0.095499]. In a stratified analysis of ethnicity, in the Mexican American, Other Hispanic, and Other Race-Including Multi-Racial groups, a significant negative correlation was found: [−0.0286 (−0.0492, −0.008)0.006593]; [−0.0295 (−0.0474, −0.0116)0.001258]; [−0.0163,(−0.0261, −0.0065)0.00114], while in the other two groups, the correlation was relatively weak (Table 2).

**TABLE 2:** The relationship between serum cotinine (ng/mL) and eGFR (mL/min).

	Model 1	Model 2	Model 3

	β (95% CI) *P*-value	β (95% CI) *P*-value	β (95% CI) *P*-value
Serum Cotinine (ng/mL)	−0.0009 (−0.0051, 0.0034) 0.681804	0.0028 (−0.0007, 0.0064) 0.117465	−0.0083 (−0.0118, −0.0047) 0.000005
**Subgroup analysis stratified by sex**			
Man	0.0021 (−0.0033, 0.0075) 0.444560	0.0059 (0.0013, 0.0105) 0.011805	−0.0048 (−0.0095, −0.0002) 0.041537
Women	−0.0041 (−0.0107, 0.0024) 0.217103	0.0002 (−0.0052, 0.0056) 0.939538	−0.0112 (−0.0166, −0.0059) 0.000039
**Subgroup analysis stratified by race/ethnicity**			
Mexican American	−0.0319 (−0.0560, −0.0079) 0.009358	−0.0140 (−0.0360, 0.0079) 0.210013	−0.0286 (−0.0492, −0.0080) 0.006593
Other Hispanic	−0.0171 (−0.0376, 0.0033) 0.100489	−0.0158 (−0.0347, 0.0031) 0.102493	−0.0295 (−0.0474, −0.0116) 0.001258
Non-Hispanic White	0.0133 (0.0076, 0.0190) 0.000005	0.0058 (0.0007, 0.0109) 0.026641	−0.0041 (−0.0093, 0.0010) 0.115603
Non-Hispanic Black	0.0031 (−0.0036, 0.0098) 0.361356	0.0048 (−0.0012, 0.0107) 0.114994	−0.0009 (−0.0070, 0.0052) 0.765077
Other Race – Including Multi-Racial	−0.0130 (−0.0239, −0.0020) 0.020253	−0.0049 (−0.0149, 0.0051) 0.334656	−0.0163 (−0.0261, −0.0065) 0.001140
**Age**			
20–39	−0.0184 (−0.0256, −0.0113) < 0.000001	−0.0036 (−0.0102, 0.0030) 0.283086	−0.0207 (−0.0275, −0.0140) < 0.000001
40–59	0.0013 (−0.0044, 0.0071) 0.653646	0.0083 (0.0029, 0.0137) 0.002481	−0.0034 (−0.0089, 0.0020) 0.221484
60–80	0.0056 (−0.0021, 0.0132) 0.154827	0.0080 (0.0005, 0.0155) 0.036988	0.0058 (−0.0010, 0.0127) 0.095499

*Model 1: no covariates were adjusted.*

*Model 2: age, sex, and race/ethnicity were adjusted.*

*Model 3: age, sex, race/ethnicity, educational background, the ratio of family poverty to income, marital status, BMI, glycosylated hemoglobin, blood glucose, BUN, triglycerides, albumin, and uric acid.*

**FIGURE 3:**
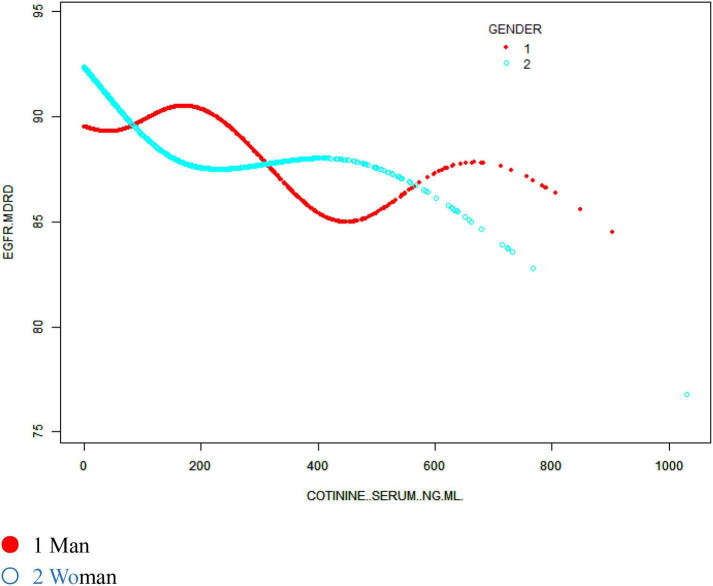
The association between serum cotinine and the eGFR stratified by sex. Age, race/ethnicity, educational background, the ratio of family poverty to income, marital status, BMI, glycosylated hemoglobin, blood glucose, BUN, triglycerides, and uric acid albumin were adjusted.

**FIGURE 4:**
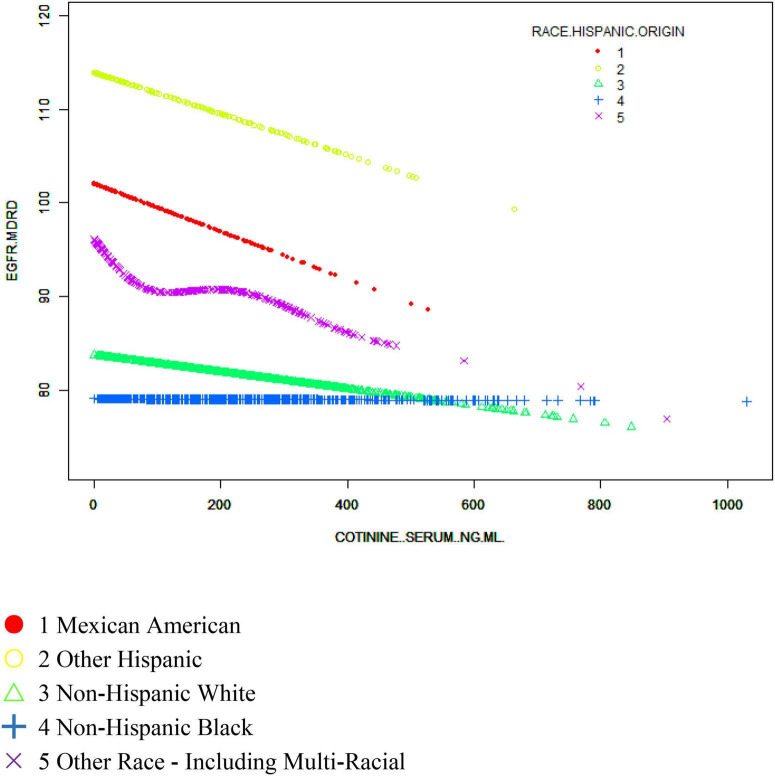
The association between serum cotinine and the eGFR stratified by race/ethnicity. Age, sex, educational background, the ratio of family poverty to income, marital status, BMI, glycosylated hemoglobin, blood glucose, BUN, triglycerides, and uric acid albumin were adjusted.

**FIGURE 5:**
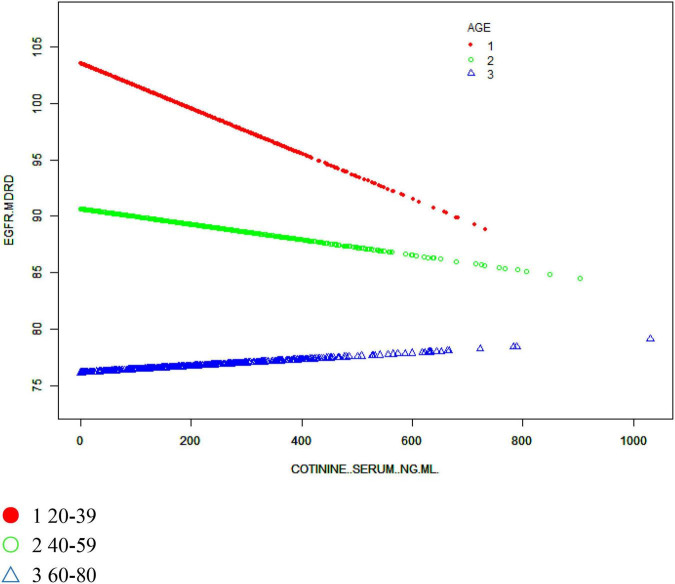
The association between serum cotinine and the eGFR stratified by age. Age, sex, educational background, the ratio of family poverty to income, marital status, BMI, glycosylated hemoglobin, blood glucose, BUN, triglycerides, and uric acid albumin were adjusted.

We also sought to identify a non-linear relationship between the eGFR and cotinine levels by smoothing curve fitting. After stratified analysis and smooth curve fitting for sex, age and race, we found inflection points at 183 and 465 ng/ml in men and at 227 and 412 ng/ml in women, and the association of the eGFR with serum cotinine levels were not significant (Figure 3). The negative association in the Mexican American and Other Hispanic groups remained evident in the stratified analysis of ethnicity but was not as significant in the Non-Hispanic White and Non-Hispanic Black groups, where the smooth curve fitting had inflection points at 137 and 262 ng/ml, respectively, and the eGFR did not significantly correlate with serum cotinine levels, but no specific conclusions regarding race were reached (Figure 4). In stratified analysis by age, serum cotinine from 20–39 years showed a clear negative association with the eGFR between 20–39 years but a weak association in those over 40 years. This result is consistent with the stratified analysis results (Figure 5).

## Discussion

Our logistic regression analysis showed a negative correlation between the eGFR and cotinine levels, which could also indirectly indicate that smoking caused a decrease in renal function. However, in specific subgroup analyses, we found different specific relationships and inflection points of 183 and 465 ng/ml for men, 227 and 412 ng/ml for women, and 137 and 262 ng/ml for other ethnic groups.

The criteria for heavy smoking were serum cotinine levels reaching 200 ng/ml (25). Our study found that the first point for both men and women was approximately 200 ng/mL (men 183 ng/ml; women 227 ng/ml). Before reaching this value, eGFR values among women decreased rapidly with increasing serum cotinine levels. After this inflection point, the eGFR demonstrated a relatively stable plateau, but the eGFR value was already below 90 ml/min, reflecting an unhealthy state, while in the population of men when the criteria of heavy smoking were reached, the eGFR decreased rapidly with increasing cotinine levels, which suggests a rapid decline in renal function. Combined with the conclusions obtained from the multiple linear regression analysis, this finding suggests that we should quit smoking as early as possible, especially in the population of men, and heavy smoking should be avoided. Through subsequent smooth curve fitting of age stratification, we found that the eGFR value of people aged 20–39 years decreased significantly with increasing cotinine levels. Despite a healthy eGFR, we should also be alert to the kidney health problems associated with increasing age, and the results of such studies also warn us that smoking cessation should be promoted among young people. At the same time, serum cotinine measurement can be used as a key to providing targeted feedback in interventions for smoking/quitting populations to promote smoking behavior changes.

The prevalence of CKD has been increasing in recent years. The 2016 epidemiological survey showed that the global prevalence of chronic kidney disease was approximately 14.3% (26), and this value has increased every year. The high prevalence, high treatment cost, and poor prognosis of CKD have seriously affected the health of people around the world and have imposed a heavy medical burden on both developed and developing countries (7). Multiple studies have been conducted on the association of second-hand smoke exposure with chronic kidney disease, with Jhee and Joo studies demonstrating a strong association of second-hand smoke with the CKD prevalence and the development of CKD (19) and an independent association with renal range proteinuria, as shown by Abiodum Omoloja et al. (20). Multiple conclusions have been drawn regarding the independent relationship between direct smoking and renal function. Isseki Maeda et al. showed that smoking was associated with an increased incidence of glomerular hyperfiltration and proteinuria (27), while a prospective cohort study in Jia Xia concluded that smoking was not associated with the incidence of proteinuria/proteinuria in the general adult population (28). However, no study has identified a non-linear relationship between smoking and renal function in different populations through stratified analysis.

In previous studies, attempts have been made to determine renal function by cotinine levels. In Charlotte Jones-Burton’s study, the use of urine cotinine as a quantitative indicator of smoking exposure in patients with CKD showed that the concentration of urine cotinine was significantly higher in smokers than in NS (29). This study demonstrates the potential utility of urine cotinine in clinical trials to examine changes in smoking behavior and its effects on kidney injury. Notably, urine cotinine is more convenient to measure, but cotinine is often metabolized in the kidney. Therefore, we cannot exclude the influence of the kidney itself. Compared with urine cotinine, serum cotinine is more accurate and intuitive. In the study of Haluk Dulger et al. serum cotinine levels were associated with renal function in active and passive smokers by three measures including the glomerular filtration rate, microalbuminuria, and β-2 microglobulin excretion to assess renal function, and cotinine levels in blood samples were collected. By comparison, the researchers found that both passive and active smoking resulted in high cotinine levels, and the serum of active smokers can show higher levels; the researchers also found that active smokers’ trace urine albumin and creatinine levels increased and concluded that the negative impact of smoking can lead to early failure of the glomeruli (30). This conclusion is consistent with the conclusion of our study. Haluk Dulger used a more comprehensive measure of renal function than that used in our study, but they did not find a relationship between smoking and nephritis or between serum cotinine and specific measures.

Regarding the specific mechanism of renal function affected by smoking, the possible mechanisms are as follows: smoking can cause chronic endothelial dysfunction, oxidative stress, and hardening of glomeruli (31, 32), while *in vivo* research evidence shows that nicotine inhalation causes the proliferation of mesangial cells (33); all the above mechanisms may lead to a decline in and deterioration of renal function. In diabetes, smoking causes renal function abnormalities that may be related to insulin resistance (34).

In our study, the results of the stratified analyses suggest a special population for the relationship between direct smoking and renal function, where the relationship between smoking and renal function is not completely linear, but multiple inflection points may exist, and the specific mechanism leading to this conclusion is still unclear. In addition, whether reducing smoking or quitting can slow CKD progression remains unknown. In the next step, we plan to use retrospective or cohort studies to determine the causal relationship between smoking and kidney disease, whether CKD progression can be delayed by reducing smoking and more accurately assess the specific kidney damage caused by smoking by adding markers.

Since we used a nationally representative sample and our sample size was sufficiently large, our results are generalizable to the entire population. However, our study still has limitations. First, this is a cross-sectional study from which we cannot derive specific causal relationships, and we need further basic mechanistic studies and related prospective cohort studies to determine the specific link between smoking and renal function. Second, given the possible impacts of kidney disease and diabetes, our study population excluded patients who may already have kidney disease and possible diabetes, which may allow us to ignore a portion of the particular population. We then used cotinine levels to assess participants’ smoking but were unable to determine the direct relationship between smoking and renal function in the particular population. Third, we cannot rule out that other confounders may have an impact on the findings.

## Conclusion

Overall, our study shows that smoking leads to a decrease in renal function in people over 20 years of age. This conclusion is particularly true in people aged 20–39 years. We hope that smoking cessation propaganda can be promoted in adolescents such that the kidney damage caused by smoking can be avoided. However, the factors leading to decreased renal function are often diverse and complex, and our study demonstrated that cigarette smoking is a risk factor for CKD.

## Data Availability Statement

The original contributions presented in this study are included in the article/Supplementary Material, further inquiries can be directed to the corresponding authors.

## Ethics Statement

The ethics review board of the National Center for Health. Statistics approved all NHANES protocols.

## Author Contributions

Y-CF wrote the manuscript, completed the drawing, and contributed to the tabulation. KX participated in the revision of the article. All authors read and approved the final manuscript.

## Conflict of Interest

The authors declare that the research was conducted in the absence of any commercial or financial relationships that could be construed as a potential conflict of interest.

## Publisher’s Note

All claims expressed in this article are solely those of the authors and do not necessarily represent those of their affiliated organizations, or those of the publisher, the editors and the reviewers. Any product that may be evaluated in this article, or claim that may be made by its manufacturer, is not guaranteed or endorsed by the publisher.

## Footnotes

1 http://www.R-project.org, The R Foundation

2 http://www.empowerstats.com X & Y Solutions, Inc., Boston, MA
